# MRI-Derived Scapular Superomedial Angle and Acromial Morphology: A Retrospective Cross-Sectional Reliability and Morphometric Association Study

**DOI:** 10.3390/jcm15176612

**Published:** 2026-08-27

**Authors:** Sami Kandefer, Volkan Gür, Mehmet Burak Gökgöz, Muhammet Ali Can, Metin Taş, Kemal Buğra Memiş, Mecit Kantarcı, Nizamettin Koçkara, Furkan Yapıcı

**Affiliations:** 1 Department of Orthopedics and Traumatology, Faculty of Medicine, Erzincan Binali Yıldırım University, 24100 Erzincan, Türkiye; skandefer@gmail.com (S.K.); drvolkangur@hotmail.com (V.G.); dr.m.burakgokgoz@gmail.com (M.B.G.); canmuhammetali93@gmail.com (M.A.C.); dr.tas.metin@gmail.com (M.T.); nzmttn@yahoo.com (N.K.); 2 Department of Radiology, Faculty of Medicine, Erzincan Binali Yıldırım University, 24100 Erzincan, Türkiye; kemalbugramemis@gmail.com (K.B.M.); akkanrad@hotmail.com (M.K.)

**Keywords:** acromion, scapula, superomedial angle, subacromial space, magnetic resonance imaging, interobserver reliability, rotator cuff

## Abstract

**Background/Objectives:** The magnetic resonance imaging (MRI)-derived scapular superomedial angle (SMA) may serve as a continuous descriptor of scapular geometry. We evaluated whether SMA is associated with acromial morphology, examined SMA and subacromial space (SAS) across acromial categories, and quantified measurement reliability. **Methods:** In this retrospective cross-sectional study, 306 shoulders from 279 patients (27 bilateral) were analyzed after screening 353 examinations. SMA and SAS were measured independently by two orthopedic observers, and acromial morphology was classified independently by two radiologists, all in blinded sessions. Reliability, group comparisons, receiver operating characteristic (ROC) analyses, and patient-level clustered generalized estimating equations were performed. **Results:** Interobserver reliability was excellent for SMA (intraclass correlation coefficient [ICC], 0.960) and SAS (ICC, 0.926), and intraobserver reliability was excellent for SMA and good for SAS (ICC, 0.944 and 0.848); acromial classification agreement was almost perfect (linear-weighted kappa, 0.980). SMA increased stepwise across Type I to III acromia (128.7°, 133.9°, 137.2°; *p* < 0.001), whereas SAS decreased (7.5, 6.8, 6.3 mm; *p* < 0.001). Each 1° SMA increase was independently associated with Type III morphology (adjusted odds ratio, 1.18; *p* < 0.001). SMA was not independently associated with rotator-cuff severity (*p* = 0.842). Because adjacent Type II–III differences approached the minimum detectable change, individual-level discrimination was limited. **Conclusions:** MRI-derived SMA is a reproducible descriptor associated with acromial morphology at the group level, but adjacent-category differences approached measurement error. SMA should not substitute for direct acromial classification, and external validation is required before clinical thresholds are considered.

## 1. Introduction

The acromion and scapular body form a complex bony framework that influences the subacromial outlet, scapulothoracic articulation, and rotator-cuff environment. Acromial morphology is conventionally classified according to the Bigliani system as Type I (flat), Type II (curved), and Type III (hooked), with a later Type IV convex variant described in magnetic resonance imaging (MRI)-based work [1,2,3]. Although this classification is widely used, its clinical and imaging reliability remain debated, and several studies have emphasized that acromial configuration should be considered alongside broader scapular and subacromial anatomy rather than as an isolated binary marker [2,4,5].

Recent morphometric studies have expanded the evaluation of acromial anatomy beyond the traditional Bigliani classification. Parameters such as the critical shoulder angle, acromion index, lateral acromial angle, and acromial coverage measurements have demonstrated associations with rotator-cuff pathology and glenohumeral joint degeneration. Nevertheless, these imaging markers show considerable overlap between symptomatic and asymptomatic individuals, suggesting that acromial morphology should be interpreted within the broader context of scapular anatomy and shoulder biomechanics rather than as an isolated determinant of disease [6,7,8,9,10,11,12].

The scapular superomedial angle (SMA), also described in the literature as the costomedial angle, reflects the geometry of the medial scapular border and the concavity of the costal surface. Previous work has primarily examined this angle in relation to snapping scapula syndrome, scapulothoracic bursitis, and osseous scapular morphology [13,14,15]. Whether this scapular parameter is linked to acromial morphology and subacromial outlet dimensions has not been sufficiently evaluated.

Scapular morphology should be considered within the broader scapulothoracic complex rather than as an isolated osseous feature. Variations in scapular anatomy, scapular positioning, and dynamic scapular kinematics may influence subacromial clearance, shoulder loading, and shoulder function [16,17,18,19,20,21,22,23,24]. Static MRI-derived measurements cannot capture dynamic biomechanics, but they can help characterize the anatomic framework within which these biomechanical processes occur.

The subacromial space (SAS) is clinically relevant because narrowing of the coracoacromial outlet may contribute to extrinsic compression of the rotator cuff, although symptoms and tendon pathology are multifactorial and cannot be explained by acromial shape alone [4,5,19]. MRI provides a practical opportunity to assess acromial morphology, scapular geometry, and soft-tissue-related subacromial dimensions within the same examination.

Accordingly, the rationale for evaluating SMA was not to replace direct acromial morphology assessment. Rather, we sought to test whether a continuous, interobserver-reliable scapular-geometry parameter could contextualize categorical acromial morphology and subacromial outlet dimensions within the same routine MRI examination, thereby providing a candidate variable for future multivariable anatomic and clinical models. It should be emphasized at the outset that SMA is not intended to substitute for direct acromial classification, and that the present study was not designed to demonstrate incremental value for predicting symptoms, rotator-cuff pathology, or treatment response; the aim was to establish whether SMA is a reproducible descriptor associated with acromial morphology, as a necessary first step before any such clinical role could be considered.

The primary objective of this study was to evaluate the association between MRI-derived SMA and acromial morphology using independent, blinded assessments of quantitative measurements and acromial classification. Secondary objectives were to examine the relationship between SMA and SAS, quantify inter- and intraobserver measurement reliability for SMA and SAS, and evaluate whether observed associations remained robust when bilateral shoulders were handled using patient-level clustering.

## 2. Materials and Methods

### 2.1. Study Design and Reporting

This retrospective, single-center, cross-sectional MRI study was prepared according to the principles of the Strengthening the Reporting of Observational Studies in Epidemiology (STROBE) statement for observational research [25] (Supplementary Material STROBE Checklist). Shoulder MR examinations performed at Erzincan University Faculty of Medicine, Mengücek Gazi Training and Research Hospital between May and June 2023 were reviewed after institutional ethics approval had been obtained.

The sample size was determined by consecutive eligibility during the predefined study period rather than by an a priori power calculation. Precision was therefore interpreted using 95% confidence intervals (CIs) around reliability and association estimates, together with the standard error of measurement and minimum detectable change for the quantitative MRI measurements.

The unit of analysis was the shoulder. Because 27 patients contributed bilateral examinations, models that could be affected by within-patient correlation were repeated using patient-level clustered generalized estimating equation (GEE) analyses.

### 2.2. Eligibility Criteria and MRI Protocol

A total of 353 shoulder MRI examinations from 325 patients were screened. Examinations were excluded for technical inadequacy, artifact, insufficient sagittal-oblique field of view, or inability to assess the scapular cross-sectional measurement plane (*n* = 20), prior acromioplasty or rotator-cuff repair (*n* = 4), healed or healing shoulder-girdle fracture (*n* = 12), or bone/soft-tissue mass in the shoulder girdle (*n* = 7). Four examinations with Type IV acromion were excluded from the primary analysis because the small number precluded reliable comparative inference and because the study hypothesis focused on the conventional Type I–III morphologic gradient. No lower age limit was applied a priori, because the acromial morphologic gradient and its relationship with the scapular superomedial angle were the primary anatomical focus; the cohort therefore included one skeletally immature adolescent (aged 15 years). To confirm that this did not influence the findings, an adult-only sensitivity analysis (patients aged ≥18 years) was pre-specified.

**Figure 1:**
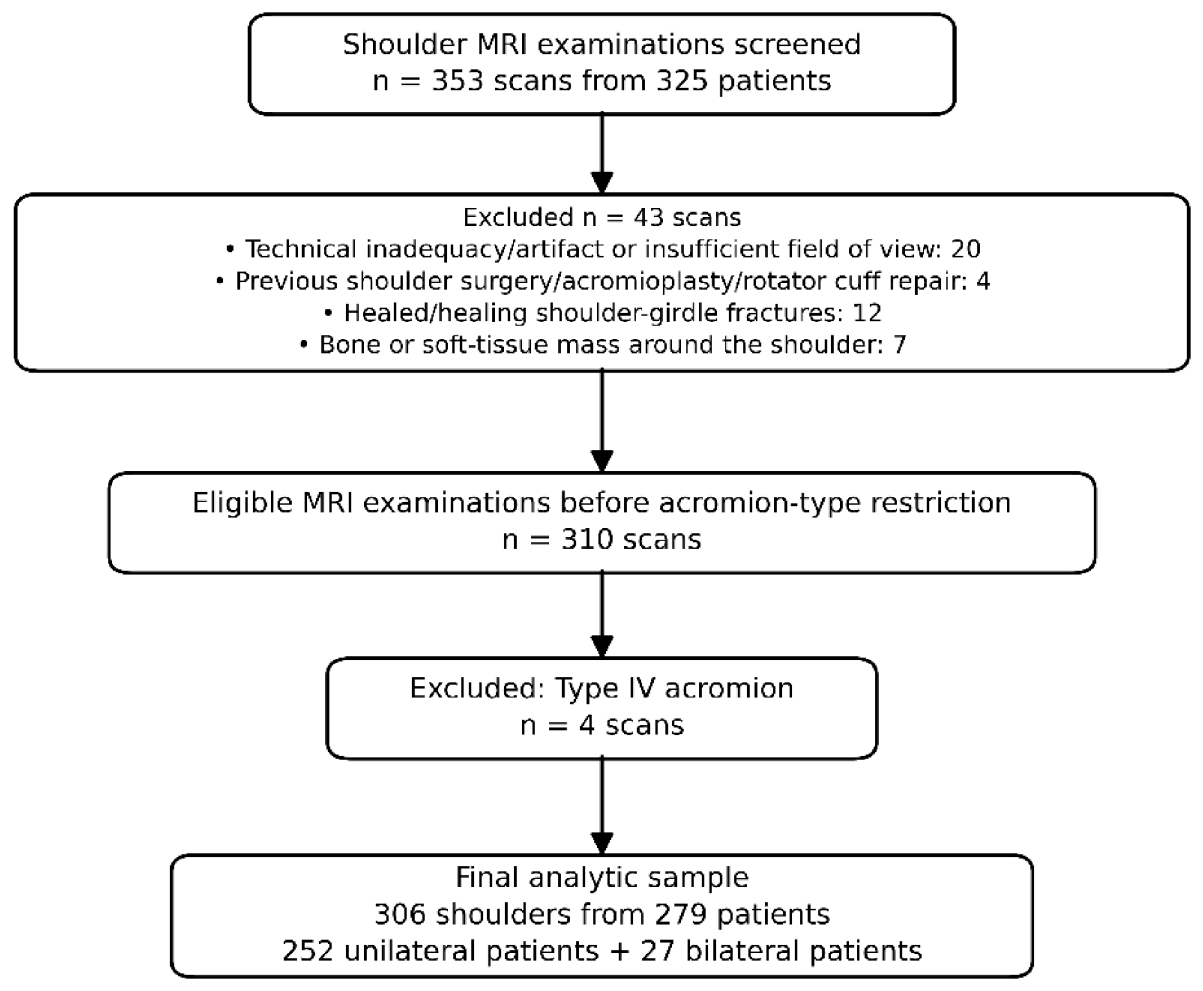
Strengthening the Reporting of Observational Studies in Epidemiology (STROBE)-style flow diagram of study selection.

The final analytic sample consisted of 306 shoulder MRIs from 279 patients (Figure 1). All examinations were performed on a 1.5-T MRI system (Magnetom Aera; Siemens Healthineers, Erlangen, Germany) using a 16-channel shoulder coil (Shoulder Shape 16; Siemens Healthineers, Erlangen, Germany). Patients were positioned supine with the examined arm at the side in slight external rotation. The shoulder protocol included coronal T1-weighted turbo spin-echo and proton-density fat-suppressed sagittal, coronal, and axial sequences. T1 coronal parameters included a 160-mm field of view, 3-mm slice thickness, repetition time of 424 ms, echo time of 11 ms, and voxel size of 0.5 × 0.5 × 3 mm. Proton-density fat-suppressed turbo spin-echo sequences used a 160-mm field of view, 2-mm slice thickness, repetition time of 2690 ms, echo time of 35 ms, and voxel size of 0.3 × 0.3 × 3 mm.

### 2.3. Image Assessment

**Figure 2:**
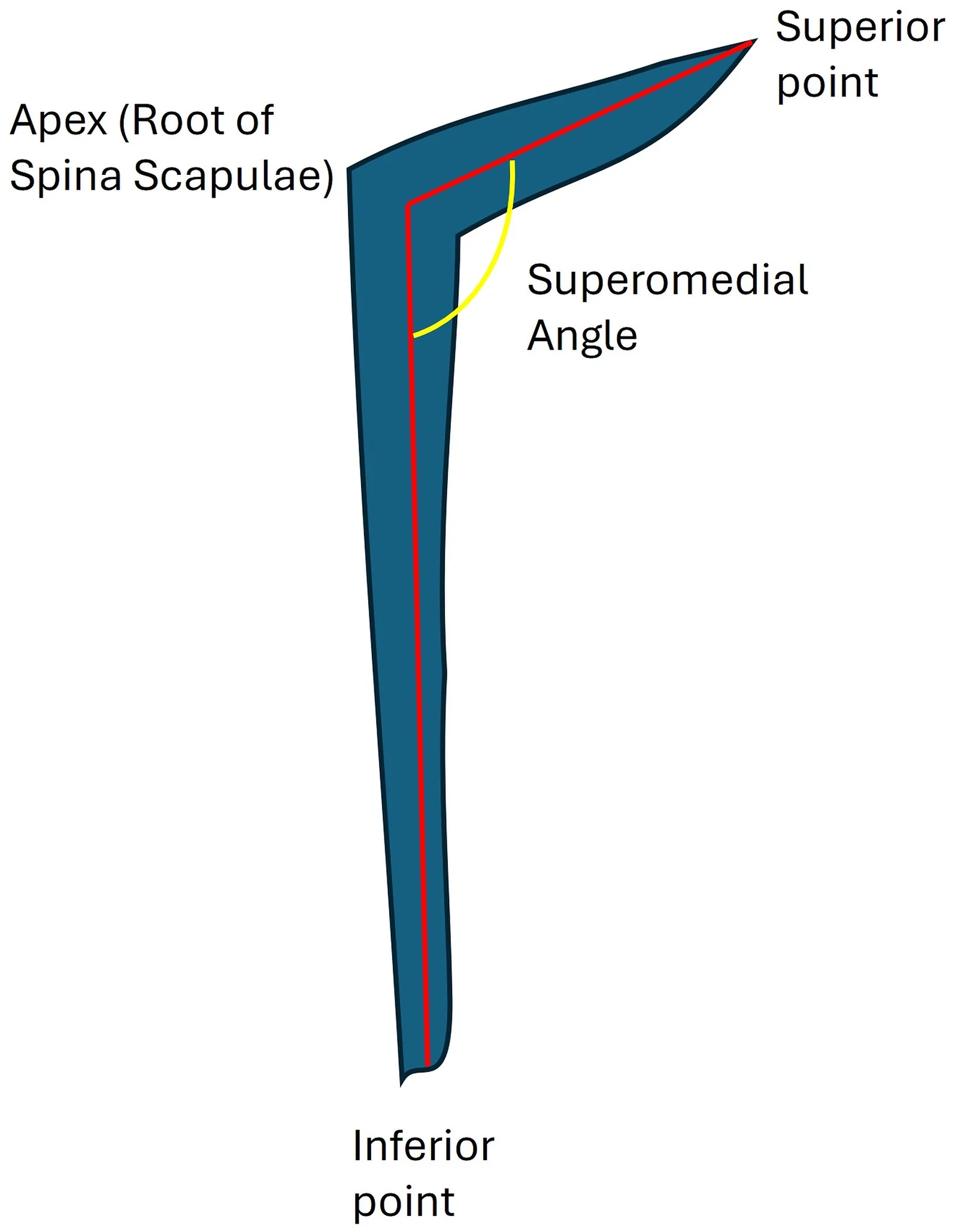
Schematic illustration of the superomedial angle (SMA) on a cross-sectional view of the scapula (medial to lateral). The angle is measured at the apex (root of the scapular spine) between the line to the superior point (most superomedial extent of the medial border) and the line to the inferior point (most distal medial-border tip).

**Figure 3:**
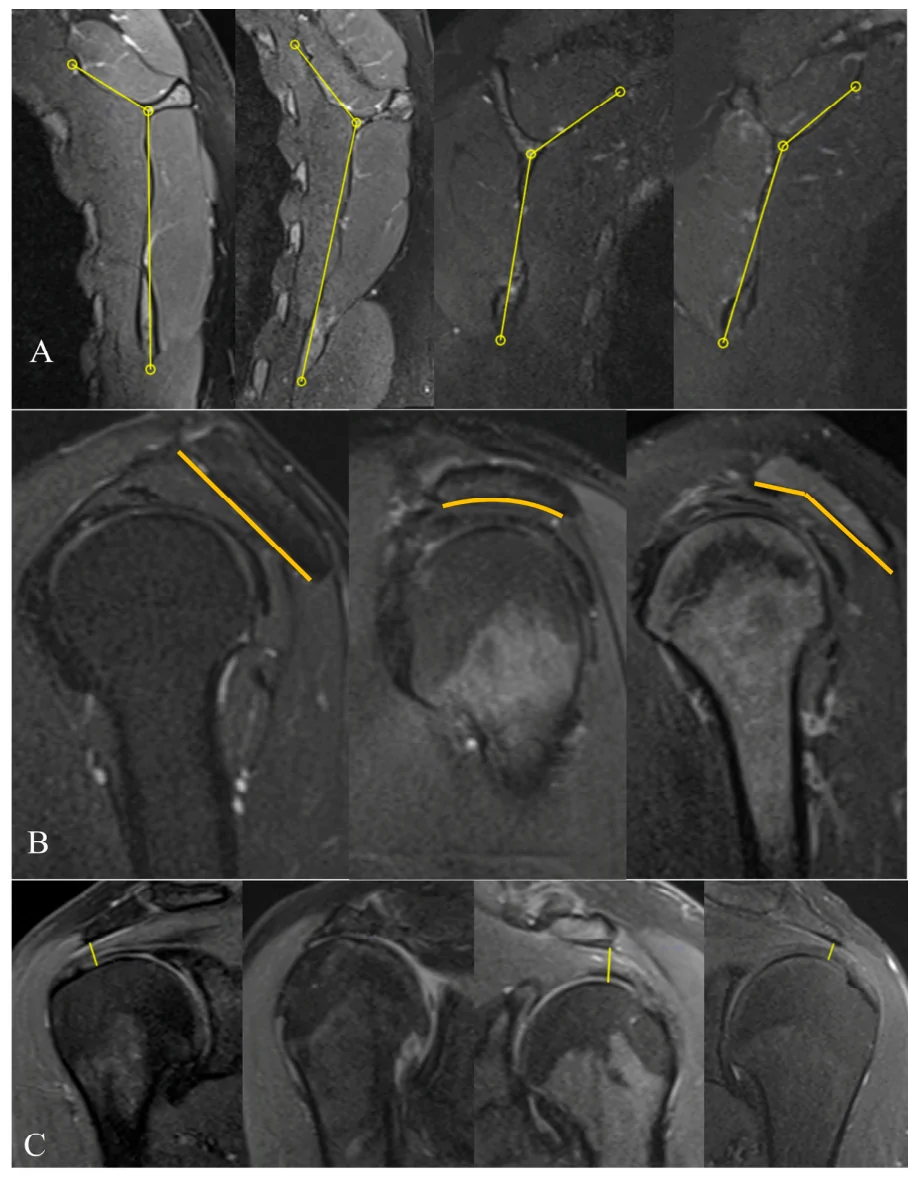
Magnetic resonance imaging (MRI)-based measurement examples: (**A**) superomedial angle (SMA), (**B**) acromial morphology, and (**C**) subacromial space (SAS). The marked lines indicate the SMA measurement in panel (**A**), the inferior acromial contour in panel (**B**), and the SAS measurement in panel (**C**).

The SMA was measured on sagittal-oblique proton-density fat-suppressed MRI sections using a predefined slice-selection algorithm. Each observer scrolled the sagittal-oblique series and selected the slice in which the medial scapular border was continuously visible over its greatest craniocaudal extent and the base of the scapular spine was clearly identifiable. If two adjacent slices were eligible, the slice with the longer uninterrupted medial border and less partial-volume artifact was selected. The superior reference point was the most superomedial point of the scapular medial border, the inferior reference point was the most distal point of the scapular medial border, and the apex of the angle was placed at the root of the scapular spine. The SMA was defined as the angle formed by two lines connecting the apex to the superior and inferior reference points (Figure 2). Measurements were performed on the institutional picture-archiving and communication system (PACS; AKGÜN PACS, version 4.1.2.22, AKGÜN Bilgisayar Program ve Hizmetleri San. Tic. A.Ş., Ankara, Türkiye) workstation using the built-in electronic angle and distance tools, with on-screen magnification standardized for each measurement and angles recorded to the nearest 1°. Adequate visualization required that the medial scapular border be captured within the sagittal-oblique field of view; examinations in which the medial border or the scapular cross-sectional measurement plane could not be fully assessed were considered technically inadequate and excluded during screening, as detailed in the eligibility criteria and Figure 1. SAS was measured on coronal-oblique sections as the shortest perpendicular distance from the inferior cortical margin of the acromion to the superior articular-cartilage margin of the humeral head, recorded to the nearest 1 mm. The coracoacromial-ligament insertion was used only as an anatomic orientation cue when visible and was not used as an alternative endpoint. Representative MRI-based measurements are shown in Figure 3. Matched slice identifiers were not available across the initial and repeat readings; therefore, slice-selection reproducibility could not be separated from landmark-placement reproducibility and was not quantified. For the primary analyses, the mean of the two quantitative observers’ measurements was used. Because all examinations were acquired on a single 1.5-T scanner with a fixed protocol, the reproducibility of SMA across field strengths, vendors, and routine shoulder MRI fields of view with smaller scapular coverage was not tested and remains to be established.

Quantitative SMA and SAS measurements and acromial classification were performed by separate sets of readers to minimize circularity and expectation bias. SMA and SAS were measured independently by two orthopedic observers: Observer 1, an orthopedic surgeon at the assistant-professor level, and Observer 2, an orthopedic specialist, each working in a separate session. Acromial morphology was classified independently by two radiologists, one at the professor level and one at the assistant-professor level, in separate blinded reading sessions. The quantitative observers did not assign acromial type, and the radiologists did not perform the SMA or SAS measurements. All sessions were performed at different times and on different workstations. The radiologists were blinded to the SMA and SAS measurements and to each other’s classifications, and the orthopedic observers were blinded to the radiologist-assigned acromial type and to each other’s measurements. Each radiologist independently classified acromial morphology while blinded to the other’s reading. For the primary analyses, a single final classification was used: the two readings were concordant in 302 of 306 shoulders, and for the four discordant shoulders, the senior radiologist’s classification was retained as the final category. The two independent radiologist readings were used to quantify interobserver reliability of acromial classification, whereas the retained final classification (Type I, 51; Type II, 178; Type III, 77) was used for all descriptive, receiver operating characteristic (ROC), generalized-estimating-equation, and supplementary sensitivity analyses.

Acromial morphology was classified on sagittal-oblique MRI by each radiologist according to the inferior acromial contour, following the Bigliani classification as adapted for MRI assessment [1,2,3]. Type I acromion was defined by a flat inferior surface. Consistent with this MRI-based operationalization, Types II and III were differentiated by locating the apex of the inferior acromial curvature relative to thirds of the anteroposterior acromial line: Type II when the apex was in the middle third, and Type III when the apex was in the anterior third. This thirds-based criterion was used as a predefined operational rule to standardize MRI reading rather than as a new classification system. The two radiologists applied this rule independently without a prior joint consensus or calibration session. MRI signs of rotator-cuff abnormality, long-head biceps abnormality, and labral tear were recorded as exploratory imaging covariates by the senior radiologist, who was blinded to the quantitative SMA and SAS measurements and assessed these variables independently of the clinical radiology report. Any rotator-cuff abnormality was defined as the presence of tendinosis, partial-thickness tear, or full-thickness (complete) tear, and rotator-cuff-normal denoted the absence of any of these findings.

### 2.4. Interobserver and Intraobserver Reliability

The two orthopedic observers independently measured SMA and SAS in separate sessions, blinded to each other’s measurements and to the radiologist-assigned acromial morphology. Interobserver reliability for the quantitative measurements was quantified using a two-way random-effects, absolute-agreement, single-measure intraclass correlation coefficient (ICC), corresponding to ICC(2,1), with 95% confidence intervals. Interobserver reliability of acromial classification between the two radiologists was assessed using the observed percentage agreement and the Cohen kappa coefficient, with linear- and quadratic-weighted kappa to account for the ordinal nature of the Bigliani categories; 95% confidence intervals for kappa were obtained by bias-corrected bootstrap resampling with 2000 replications. Reliability interpretation followed commonly used thresholds: <0.50 poor, 0.50–0.75 moderate, 0.75–0.90 good, and >0.90 excellent [26,27]. Bland–Altman analysis was used to estimate systematic bias and 95% limits of agreement (LoA). The standard error of measurement (SEM) and the minimum detectable change at the 95% confidence level (MDC95) were also calculated.

The methodological approach for reliability assessment was based on established recommendations for the interpretation of intraclass correlation coefficients and agreement analysis. ICC estimates were complemented by Bland–Altman analysis to evaluate measurement agreement and potential systematic bias, consistent with current recommendations for quantitative imaging research [28,29,30]. Intraobserver reliability was additionally evaluated: Observer 1 repeated the SMA and SAS measurements in all 306 shoulders after a washout interval of at least six weeks, blinded to the initial values. Intraobserver reliability was quantified using a two-way mixed-effects, absolute-agreement, single-measure ICC, with the same Bland–Altman analysis, SEM, and MDC95 framework. Bland–Altman bias was defined consistently as the first listed measurement minus the second listed measurement, and SEM and MDC95 were derived from the corresponding absolute-agreement estimates.

### 2.5. Statistical Analysis

Continuous variables were summarized using mean ± standard deviation and median with interquartile range (IQR). Categorical variables were summarized as counts and percentages. Distributional assumptions were evaluated using visual inspection and normality tests. Between-group comparisons for continuous variables were performed using Kruskal–Wallis tests with post-hoc Mann–Whitney U tests and Holm correction where appropriate. Categorical variables were compared using chi-square or Fisher’s exact tests. The direct relationship between SMA and SAS was assessed using Pearson and Spearman correlation coefficients, both in the overall cohort and within each acromial morphology category, and supplemented by simple linear regression of SAS on SMA.

ROC analyses were used only to describe the ability of SMA to discriminate acromial morphology in pairwise comparisons. Areas under the curve (AUCs) were generated from the IBM SPSS Statistics ROC procedure and reported with bootstrap 95% confidence intervals. Sample-derived thresholds were identified using the Youden index for exploratory description; sensitivity, specificity, positive predictive value (PPV), and negative predictive value (NPV) are reported in the Supplementary Materials as sample-prevalence-dependent metrics and were not considered externally generalizable diagnostic values. To estimate internal optimism, threshold-independent discrimination (AUC) was additionally evaluated using bootstrap resampling with 2000 replications.

To account for bilateral shoulder clustering, GEE logistic regression with an exchangeable working correlation structure was used for Type III versus Type I/II and Type I versus Type II/III outcomes. These binary models were used to evaluate the morphologic extremes of the conventional Type I–III spectrum under patient-level clustering. These clustered binary GEE models were pre-specified as the primary inferential analysis, because they directly address the two clinically meaningful contrasts (the presence of a Type III hooked acromion and the presence of a flat Type I acromion) while providing valid population-averaged estimates under bilateral-shoulder clustering, which the ordinal model accommodates less transparently. Covariates included SMA, SAS, age, sex, and side. Because acromial morphology is intrinsically ordinal, a proportional-odds ordinal logistic regression of acromial type on SMA and SAS was additionally fitted, both without clustering and with cluster-robust (sandwich) standard errors accounting for patient-level clustering, and is reported in the Supplementary Materials. In an additional analysis, rotator-cuff status was modeled as a four-level ordinal outcome (normal, tendinosis, partial-thickness tear, full-thickness tear) using ordinal logistic regression with patient-level cluster-robust (sandwich) standard errors, and as a binary outcome (any abnormality versus normal) using generalized estimating equations, in both cases adjusting for SMA, SAS, age, sex, and acromial type, to assess whether SMA carried independent information about rotator-cuff disease. Consistent with this hierarchy, the proportional-odds model was treated as a supporting analysis because the direction of association was consistent across cut-points but some variation in coefficient magnitude was observed, and the pairwise ROC analyses were regarded as exploratory descriptions of discriminative ability rather than inferential tests. An additional SAS-outlier sensitivity analysis was performed after excluding shoulders with an observer-mean SAS < 3 mm to evaluate whether extremely low subacromial-space values influenced the group-level gradients or clustered multivariable associations. A two-sided *p*-value < 0.05 was considered statistically significant. There were no missing values for any variable included in the primary or sensitivity analyses. Statistical analyses were performed using IBM SPSS Statistics, version 30.0 (IBM Corp., Armonk, NY, USA), and R statistical software version 4.3.1 (R Foundation for Statistical Computing, Vienna, Austria). Descriptive statistics, group comparisons, ROC analyses, and conventional regression outputs were generated in SPSS; correlation analyses, ordinal logistic regression, cluster-robust sensitivity analyses, and bootstrap-based confidence intervals or sensitivity procedures were performed or verified in R.

## 3. Results

### 3.1. Cohort Characteristics

Among 353 screened shoulder MRI examinations, 306 shoulders from 279 patients met the final analytic criteria. Twenty-seven patients contributed bilateral shoulders. The mean age was 48.0 ± 12.0 years, and 180 shoulders (58.8%) were from female patients. Right-sided examinations accounted for 157 shoulders (51.3%). Acromial morphology was Type I in 51 shoulders (16.7%), Type II in 178 (58.2%), and Type III in 77 (25.2%).

Using the observer-mean measurement, SMA ranged from 119.0° to 149.5° with a mean of 133.9 ± 5.6° and median of 133.5° (IQR, 130.5–137.5°). SAS ranged from 1.0 to 11.0 mm with a mean of 6.8 ± 1.3 mm. Baseline demographic and imaging characteristics are provided in Table 1.

### 3.2. Interobserver and Intraobserver Reliability

**Figure 4:**
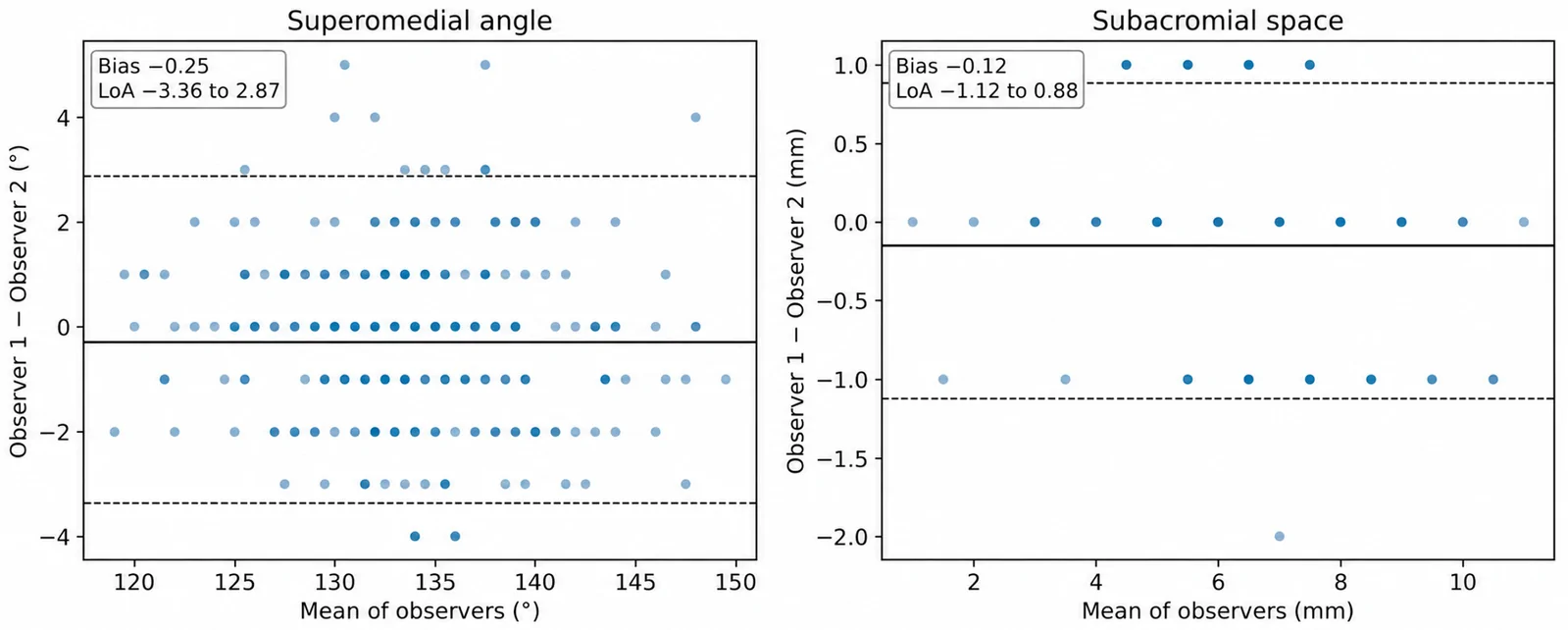
Bland–Altman plots for interobserver agreement in superomedial-angle and subacromial-space measurements. In both Bland–Altman plots, each point represents one shoulder; darker blue indicates overlapping observations with identical plotted values. The solid horizontal line indicates the mean bias (Observer 1 minus Observer 2), and the dashed horizontal lines indicate the 95% limits of agreement (LoA).

Inter- and intraobserver reliability estimates are summarized in Table 2, and the interobserver Bland–Altman plots are shown in Figure 4. Interobserver reliability was excellent for both quantitative MRI measures. For SMA, ICC was 0.960 (95% CI, 0.950–0.968), with a mean observer difference of −0.25° and 95% LoA from −3.36° to 2.87°. For SAS, ICC was 0.926 (95% CI, 0.905–0.942), with a mean observer difference of −0.12 mm and 95% LoA from −1.12 to 0.88 mm. Intraobserver reliability was excellent for SMA (ICC, 0.944; 95% CI, 0.930–0.955) and good for SAS (ICC, 0.848; 95% CI, 0.813–0.877), with no meaningful systematic drift (SMA bias, −0.01°, 95% LoA −3.83° to 3.80°; SAS bias, −0.02 mm, 95% LoA −1.57 to 1.52 mm). Repeat SAS measurements were analyzed at the same 1-mm resolution as the initial measurements. The intraobserver MDC95 was 3.82° for SMA and 1.54 mm for SAS. Acromial classification showed almost perfect agreement between the two radiologists, with 302 of 306 shoulders identically classified (98.7% agreement). The Cohen kappa was 0.977 (95% CI, 0.952–0.995), with a linear-weighted kappa of 0.980 (95% CI, 0.960–0.995) and a quadratic-weighted kappa of 0.984. All four discordant classifications involved adjacent categories (one Type I versus Type II and three Type II versus Type III); no Type I versus Type III disagreement occurred. For these four shoulders, the senior radiologist’s classification was retained as the final category used in all subsequent analyses.

### 3.3. Association Between SMA, SAS, and Acromial Morphology

SMA showed a stepwise increase across acromial morphology groups: Type I, 128.7 ± 4.2°; Type II, 133.9 ± 5.4°; and Type III, 137.2 ± 4.4° (*p* < 0.001). Post-hoc testing showed significant differences between all pairwise morphologic categories after Holm correction. Conversely, SAS decreased across the same morphologic gradient: Type I, 7.5 ± 1.2 mm; Type II, 6.8 ± 1.3 mm; and Type III, 6.3 ± 1.2 mm (*p* < 0.001). Although SMA and SAS showed opposite trends across acromial morphology categories, they were not directly correlated with each other. The overall correlation between SMA and SAS was negligible (Pearson r = −0.03, *p* = 0.607; Spearman rho = −0.10, *p* = 0.095), and within-type correlations were similarly weak and non-significant (Type I, r = 0.12; Type II, r = 0.14; Type III, r = 0.07; all *p* > 0.05). Linear regression confirmed the absence of a meaningful linear relationship (beta = −0.007 mm per degree; *p* = 0.607; R-squared < 0.001). These correlation results are detailed in Supplementary Table S2. The opposing group-level trends therefore reflect parallel associations with acromial morphology rather than a direct inverse relationship between the two measurements at the individual level. An additional outlier sensitivity analysis excluding three shoulders with observer-mean SAS < 3 mm (all Type II acromia and all with full-thickness rotator-cuff tears) preserved the stepwise SMA and SAS gradients and the direction and statistical significance of the clustered multivariable associations (Supplementary Table S3).

Age differed modestly across acromion types (Kruskal–Wallis *p* = 0.036), driven mainly by a higher age in Type III compared with Type II (Holm-corrected post-hoc *p* = 0.041). Sex and side distributions did not differ significantly across acromion types. Exploratory analysis showed that any rotator-cuff abnormality was more frequent in Type II and Type III groups than in Type I; however, this retrospective MRI-indication-based finding should not be interpreted causally (Table 3). When these between-category differences were compared directly with the measurement error, the adjacent Type II versus Type III SMA difference (+3.3°) fell below the intraobserver MDC95 (3.82°), and most SAS differences were within their corresponding MDC95, indicating that adjacent-category separation is limited at the individual level (Table 4). These gradients were robust in an adult-only subgroup (*n* = 305): the stepwise SMA increase (Type I, 128.7°; Type II, 133.8°; Type III, 137.2°) and SAS decrease (7.5, 6.8, and 6.3 mm, respectively) were essentially identical to those in the full cohort (both *p* < 0.001), confirming that inclusion of the single adolescent aged 15 years did not drive the associations.

### 3.4. ROC Analyses and Threshold Findings

**Figure 5:**
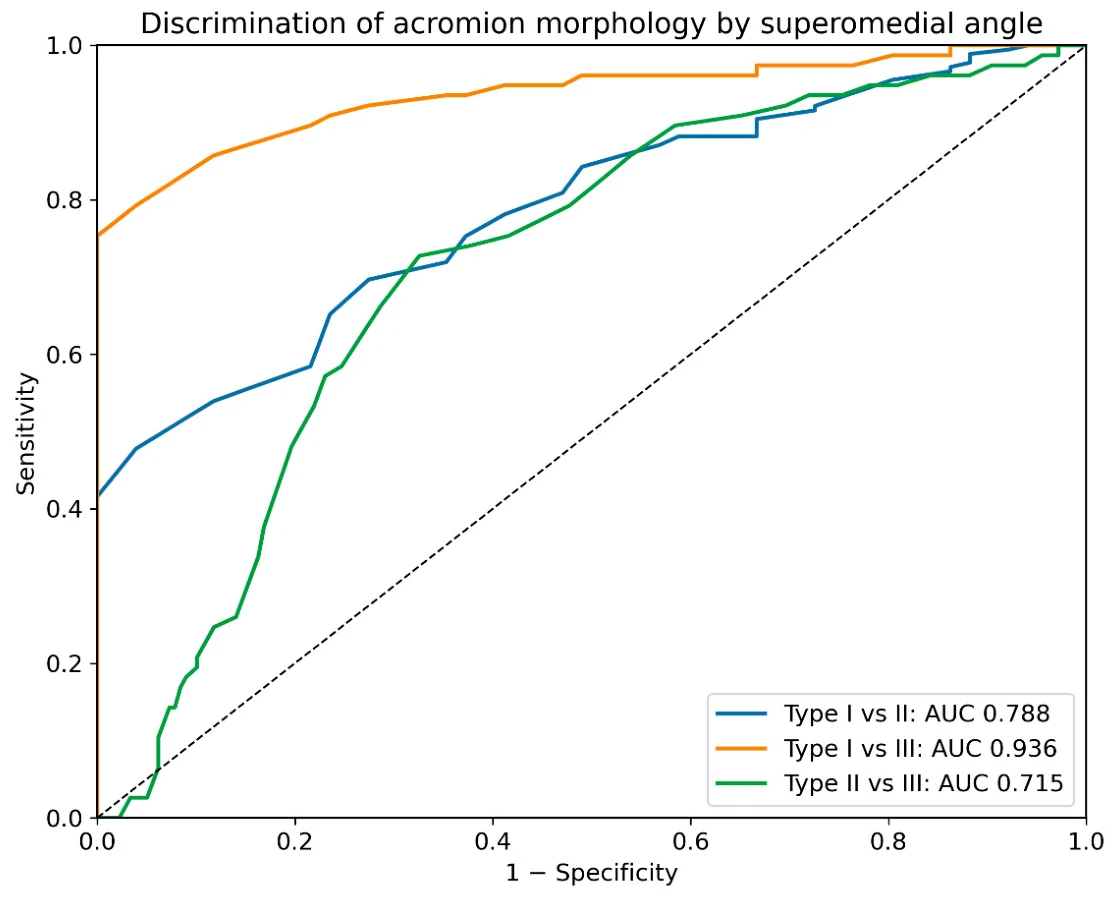
Receiver-operating-characteristic curves for superomedial angle in pairwise acromial morphology discrimination. The dashed diagonal line represents the line of no discrimination, corresponding to an area under the curve (AUC) of 0.5.

ROC analyses were performed to characterize, in exploratory fashion, how strongly the continuous SMA tracked the categorical acromial gradient, not to derive clinically applicable cutoffs. SMA discriminated acromion morphologies with the highest accuracy for the most morphologically distinct comparison, Type I versus Type III (AUC, 0.936; 95% CI, 0.891–0.972). Discrimination was moderate for Type I versus Type II (AUC, 0.788; 95% CI, 0.724–0.848) and Type II versus Type III (AUC, 0.715; 95% CI, 0.647–0.779). Pairwise AUC values are summarized in Table 5, and the corresponding ROC curves are shown in Figure 5. Optimism-corrected AUC estimates are provided in Supplementary Table S4. Sample-derived Youden thresholds and their corresponding sensitivity, specificity, and prevalence-dependent predictive values are provided in Supplementary Table S5; these thresholds are exploratory only and should not be interpreted as clinical decision cutoffs without external validation.

### 3.5. Cluster-Robust Multivariable Analyses

In the GEE model accounting for patient-level clustering, higher SMA and lower SAS remained independently associated with Type III morphology after adjustment for age, sex, and side. A supporting ordinal proportional-odds model with cluster-robust standard errors showed the same pattern (Supplementary Table S6). Each 1° increase in SMA increased the odds of Type III morphology by 18% (odds ratio [OR], 1.18; 95% CI, 1.11–1.25; *p* < 0.001), while each 1-mm increase in SAS decreased the odds by 33% (OR, 0.67; 95% CI, 0.52–0.86; *p* = 0.002). Age, sex, and side were not statistically significant after adjustment. In the complementary Type I model, higher SMA was inversely associated with Type I morphology, whereas larger SAS was positively associated with Type I morphology (Table 6).

### 3.6. Association Between SMA and Rotator-Cuff Status

To evaluate whether SMA carried independent clinical information beyond its structural association with acromial morphology, rotator-cuff status was modeled as an ordinal outcome (normal, tendinosis, partial-thickness tear, full-thickness tear) using ordinal logistic regression with patient-level cluster-robust (sandwich) standard errors, adjusting for SMA, SAS, age, sex, and acromial type. SMA was not independently associated with rotator-cuff severity (OR, 1.00 per degree; 95% CI, 0.96–1.06; *p* = 0.842). In contrast, smaller SAS and older age were independently associated with greater rotator-cuff severity (SAS: OR, 0.61 per millimeter; 95% CI, 0.48–0.78; *p* < 0.001; age: OR, 1.07 per year; 95% CI, 1.05–1.10; *p* < 0.001), whereas sex and acromial type were not (Table 7). A binary model (any rotator-cuff abnormality versus normal) using generalized estimating equations showed the same pattern.

## 4. Discussion

This retrospective MRI study provides three main findings. First, SMA and SAS demonstrated excellent interobserver reliability when the medial scapular border was adequately included within the sagittal-oblique MRI field of view. Second, SMA showed a stepwise group-level increase from Type I to Type III acromial morphology, whereas SAS decreased across the same morphologic gradient. Third, SMA and SAS were not directly correlated at the individual level, indicating that they represent parallel associations with acromial morphology rather than a simple inverse relationship between the two measurements.

The measurement protocol was designed to minimize circularity between the quantitative predictor and the morphologic outcome. Acromial morphology was classified independently by two radiologists in blinded sessions, whereas SMA and SAS were measured by two separate orthopedic observers who were blinded to acromial type. Therefore, the observed SMA-acromion association was not generated by a single assessor simultaneously measuring the angle and assigning the acromial category.

The reliability results should be interpreted precisely. The ICC values for SMA and SAS exceeded 0.90, and Bland–Altman limits of agreement were narrow relative to the full observed measurement range, supporting excellent interobserver agreement in this cohort. Intraobserver repeatability was excellent for SMA and good for SAS (ICC 0.944 and 0.848, respectively); however, the intraobserver MDC95 for SMA (3.82°) exceeded the mean difference between adjacent Type II and Type III acromia, indicating that a single reader’s measurement error alone can approach or exceed adjacent-category differences. In addition, SMA measurement depends not only on drawing the landmarks but also on selecting the appropriate sagittal-oblique slice. Matched slice identifiers were not available across the initial and repeat readings; therefore, slice-selection repeatability could not be evaluated separately from landmark-placement repeatability. These factors should be tested in dedicated repeat-measurement and multicenter studies before SMA is considered a fully validated scapular-geometry descriptor.

The relationship between measurement error and between-group differences is central to interpretation. The interobserver MDC95 for SMA was approximately 3.12°, and the adjacent Type II versus Type III SMA difference (3.3°) exceeded this value only slightly while remaining below the intraobserver MDC95 of 3.82°. In other words, even the adjacent-group difference that exceeded interobserver measurement error did so marginally and was smaller than within-observer repeat-measurement error. Likewise, the interobserver MDC95 for SAS was approximately 1.0 mm and the intraobserver MDC95 was 1.54 mm, whereas the mean SAS differences between adjacent categories were smaller still. Thus, the statistically robust group-level gradients should not be translated into individual-patient classification. The ROC analyses should be read in the same light. Although SMA separated the most distinct categories well (Type I versus Type III, AUC 0.936), discrimination for the clinically more demanding Type II versus Type III comparison was only moderate (AUC 0.715), consistent with a between-category difference that lies within the limits of measurement error. The ROC results are therefore best regarded as an exploratory description of how the continuous SMA maps onto acromial morphology, and the sample-derived Youden thresholds should not be adopted as diagnostic cutoffs; deriving separate cutoffs for adjacent categories is not warranted in the absence of external validation. SMA is best interpreted as a continuous scapular-geometry descriptor rather than as a surrogate for Bigliani classification.

The almost perfect interradiologist agreement for acromial classification also requires context. A weighted kappa of 0.980 indicates that the outcome was categorized reproducibly in this single-center dataset, but this value is higher than the reliability commonly reported for Bigliani typing, which has ranged from only fair to moderate interobserver agreement in several prior studies [11,12]. The high agreement in the present cohort likely reflects the fixed MRI protocol, experienced readers, and the predefined thirds-based operational rule used to distinguish Type II from Type III morphology. The original Bigliani system was described on supraspinatus-outlet radiographs and relies on the overall shape of the inferior acromial surface (flat, curved, or hooked), whereas our thirds-based rule operationalizes the same qualitative gradient by fixing the anteroposterior location of the curvature apex. This reduces borderline Type II versus Type III ambiguity and probably explains the higher agreement; to our knowledge, this specific MRI adaptation has not been independently validated against the original radiographic classification. This rule improved standardization but should be viewed as an MRI-based operationalization of Bigliani morphology rather than a separately validated classification system. External validation in less standardized multireader environments is necessary.

The potential role of SMA should therefore be interpreted as complementary rather than substitutive. Direct acromial classification remains necessary when the inferior acromial contour is the question of interest. SMA adds a continuous and interobserver-reliable scapular-geometry variable that may be useful for describing the broader scapular framework, contextualizing borderline morphology, and supporting future multivariable models that integrate acromial type, subacromial dimensions, and clinical outcomes. The present study does not show that SMA improves prediction of symptoms, rotator-cuff pathology, or treatment response.

SAS should be interpreted as a secondary anatomic observation. Although SAS decreased across acromial morphology groups and remained associated with Type III morphology in clustered multivariable models, static SAS can be influenced by many factors not separately modeled here, including acromial spur, acromioclavicular-joint osteoarthritis, subacromial osteophytes, os acromiale, superior humeral-head migration, tendon bulk, effusion, and rotator-cuff tear size or retraction. Sensitivity analyses showed that the SMA and SAS gradients persisted after exclusion of full-thickness rotator-cuff tears (Supplementary Table S7), within rotator-cuff-normal and rotator-cuff-abnormal subgroups (Supplementary Table S8), and across age strata (Supplementary Table S9), and in an adult-only subgroup (Supplementary Table S10), and remained consistent after exclusion of the three shoulders with observer-mean SAS < 3 mm. Furthermore, within each acromion type, no statistically significant association between SMA and recorded cuff severity was detected, and the exploratory Type II versus Type III thresholds were broadly similar in cuff-normal and cuff-abnormal shoulders (Supplementary Table S11). However, these analyses were based on small subgroup cells and did not include a formal interaction test, and they do not exclude residual confounding by rotator-cuff degeneration, acromial or subacromial osteophytes, acromioclavicular-joint degeneration, os acromiale, shoulder positioning, or other unmeasured variables. Accordingly, the findings indicate only that SMA was not independently associated with the recorded cuff-severity variable in this cohort; they do not establish that SMA is degeneration-independent or biomechanically fixed. Because SAS, unlike SMA, was independently associated with rotator-cuff severity, part of the observed SAS-morphology relationship may reflect secondary degenerative narrowing rather than a purely structural morphometric association.

The relationship between SMA and rotator-cuff status further clarifies what SMA does and does not represent. After adjustment for SAS, age, sex, and acromial type, SMA was not independently associated with rotator-cuff severity, whereas smaller SAS and older age were. This finding supports only that SMA should not be interpreted as a direct marker of rotator-cuff severity in this cohort; it does not exclude unmeasured degenerative, positional, or imaging-related influences on SMA. The associations of smaller SAS and older age with cuff severity are compatible with a contribution from secondary degenerative narrowing. Overall, SMA is best regarded as a reproducible morphometric parameter associated with acromial morphology, and should not be interpreted as a standalone predictor of cuff pathology. The independent association of SAS and age with cuff severity is expected, since narrower subacromial space and advancing age are established correlates of degenerative rotator-cuff change, and is compatible with SAS reflecting a greater contribution from secondary degenerative narrowing than was captured by the SMA association in this cohort.

The explicit handling of bilateral shoulders is a methodological strength. Treating both shoulders from the same patient as fully independent can underestimate standard errors; therefore, clustered GEE models were used and confirmed that higher SMA and lower SAS remained associated with Type III morphology after adjustment for age, sex, and side. Because acromial morphology is ordinal, an ordinal proportional-odds model with cluster-robust standard errors was also fitted as a supporting analysis. The direction of association was consistent across ordinal cut-points, but some variation in coefficient magnitude was observed, so the ordinal model was used to support rather than replace the primary clustered binary models.

The study also has several limitations. It was retrospective and single-center, and MRI examinations were obtained for clinical indications rather than from asymptomatic volunteers. Because imaging was clinically indicated, roughly three-quarters of the shoulders showed some degree of rotator-cuff abnormality, so the observed distribution of acromial morphology and the SMA and SAS relationships may over-represent symptomatic, cuff-affected shoulders and may not generalize to an asymptomatic or population-based sample. The morphometric associations reported here should therefore be regarded as internally consistent within a symptomatic referral population rather than as normative values, and external validation in unselected cohorts is required before the observed gradients can be generalized. Standardized physical examination, pain and function scores, symptom duration, trauma history, hand dominance, occupational exposure, dynamic scapular-motion assessment, and intraoperative confirmation were not available. Degenerative and positional variables that may affect the subacromial space, such as subacromial or acromial osteophytes, acromioclavicular-joint osteoarthritis, os acromiale, and shoulder positioning, were not separately graded, so their potential influence on the SMA thresholds could not be modeled directly; the analysis of rotator-cuff severity (Supplementary Table S11) addresses only the degenerative dimension that was systematically recorded. Type IV acromion was excluded because only four such examinations were identified, so the findings apply only to the conventional Type I–III spectrum. Future studies should test slice-selection stability, multicenter interscanner reproducibility, three-dimensional scapular morphology, dynamic scapulothoracic motion, and whether SMA provides incremental predictive value beyond established acromial and subacromial parameters for clinically meaningful outcomes. In particular, prospective longitudinal cohorts will be required to determine whether SMA carries any independent prognostic value for the development or progression of rotator-cuff pathology, since the present cross-sectional design cannot establish a temporal or causal relationship.

## 5. Conclusions

MRI-derived scapular superomedial angle demonstrated excellent interobserver and intraobserver reliability and a stepwise group-level association with acromial morphology in this retrospective clinical MRI cohort. SMA increased while SAS decreased across Type I–III acromial morphology groups; however, SMA and SAS were not directly correlated at the individual level, indicating parallel group-level trends rather than a direct inverse relationship. The adjacent Type II versus Type III SMA difference exceeded the interobserver MDC95 only slightly and remained below the intraobserver MDC95, and slice-selection repeatability could not be separated from landmark-placement repeatability. Therefore, SMA should be regarded as a continuous morphometric descriptor of scapular geometry rather than a substitute for direct acromial classification or an individual-level diagnostic threshold. Prospective multicenter, multireader, and interscanner validation is required before diagnostic or therapeutic thresholds are adopted.

## Figures and Tables

**Table 1:** Baseline imaging and demographic characteristics of the analytic cohort.

Characteristic	Value
Shoulders, *n*	306
Patients, *n*	279
Unilateral/bilateral patients	252/27
Age, years	48.0 ± 12.0; median 50.0 (40.0–58.0); range 15–80
Sex: female	180 (58.8%)
Sex: male	126 (41.2%)
Side: right	157 (51.3%)
Side: left	149 (48.7%)
Acromion type: Type I	51 (16.7%)
Acromion type: Type II	178 (58.2%)
Acromion type: Type III	77 (25.2%)
Rotator cuff: normal	76 (24.8%)
Rotator cuff: tendinosis	97 (31.7%)
Rotator cuff: partial tear	115 (37.6%)
Rotator cuff: full-thickness tear	18 (5.9%)
Long-head biceps: normal	190 (62.1%)
Long-head biceps: tendinitis	108 (35.3%)
Long-head biceps: rupture	8 (2.6%)
Labrum: normal	235 (76.8%)
Labrum: tear	71 (23.2%)
Superomedial angle, ° (observer mean)	133.9 ± 5.6; median 133.5 (130.5–137.5); range 119.0–149.5
Subacromial space, mm (observer mean)	6.8 ± 1.3; median 7.0 (6.0–7.5); range 1.0–11.0

**Table 2:** Inter- and intraobserver reliability for superomedial angle and subacromial space. Interobserver agreement for acromial classification between the two radiologists is reported in the text and in Supplementary Table S1.

**Interobserver**								
**Measurement**	**Observer 1**	**Observer 2**	**ICC**	**95% CI**	**Bias**	**95% LoA**	**SEM**	**MDC95**
Superomedial angle (°)	133.74 ± 5.66	133.99 ± 5.75	0.960	0.950–0.968	−0.25	−3.36 to 2.87	1.125	3.120
Subacromial space (mm)	6.72 ± 1.31	6.84 ± 1.40	0.926	0.905–0.942	−0.12	−1.12 to 0.88	0.362	1.002
**Intraobserver**								
**Measurement**	**Reading 1**	**Reading 2**	**ICC**	**95% CI**	**Bias**	**95% LoA**	**SEM**	**MDC95**
Superomedial angle (°)	133.74 ± 5.66	133.75 ± 5.95	0.944	0.930–0.955	−0.01	−3.83 to 3.80	1.38	3.82
Subacromial space (mm)	6.72 ± 1.31	6.74 ± 1.53	0.848	0.813–0.877	−0.02	−1.57 to 1.52	0.56	1.54

Bias was calculated as the first listed measurement minus the second listed measurement (Observer 1 minus Observer 2 for interobserver analysis; Reading 1 minus Reading 2 for intraobserver analysis). For the intraobserver analysis, Reading 1 and Reading 2 refer to Observer 1’s initial and repeat measurements, respectively. Repeat subacromial space measurements were rounded to the protocol-defined 1-mm resolution using the conventional round-half-up method before analysis. ICC, intraclass correlation coefficient; CI, confidence interval; LoA, limits of agreement; SEM, standard error of measurement; MDC95, minimum detectable change at the 95% confidence level.

**Table 3:** Characteristics according to acromial morphology.

Variable	Type I (*n* = 51)	Type II (*n* = 178)	Type III (*n* = 77)	*p*-Value
Age, years	47.0 ± 11.7; 49.0 (42.0–53.5)	46.9 ± 12.2; 49.0 (39.0–55.8)	51.2 ± 11.5; 52.0 (45.0–60.0)	0.036
SMA, °	128.7 ± 4.2; 129.0 (126.0–132.0)	133.9 ± 5.4; 133.5 (131.0–136.0)	137.2 ± 4.4; 137.5 (134.5–139.5)	<0.001
SAS, mm	7.5 ± 1.2; 7.5 (7.0–8.0)	6.8 ± 1.3; 7.0 (6.0–7.5)	6.3 ± 1.2; 6.0 (6.0–7.0)	<0.001
Female sex	26 (51.0%)	115 (64.6%)	39 (50.6%)	0.053
Right side	32 (62.7%)	84 (47.2%)	41 (53.2%)	0.136
Any rotator-cuff abnormality	31 (60.8%)	136 (76.4%)	63 (81.8%)	0.022
Any long-head biceps abnormality	16 (31.4%)	65 (36.5%)	35 (45.5%)	0.231
Labral tear	8 (15.7%)	49 (27.5%)	14 (18.2%)	0.102

SMA, superomedial angle; SAS, subacromial space.

**Table 4:** Between-category differences in SMA and SAS relative to measurement error (MDC95).

Comparison	Mean Difference	Interobserver MDC95	Intraobserver MDC95	Exceeds Interobserver MDC95?	Exceeds Intraobserver MDC95?
SMA, Type I vs. II	+5.2°	3.12°	3.82°	Yes	Yes
SMA, Type II vs. III	+3.3°	3.12°	3.82°	Yes	No
SMA, Type I vs. III	+8.6°	3.12°	3.82°	Yes	Yes
SAS, Type I vs. II	−0.7 mm	1.00 mm	1.54 mm	No	No
SAS, Type II vs. III	−0.5 mm	1.00 mm	1.54 mm	No	No
SAS, Type I vs. III	−1.2 mm	1.00 mm	1.54 mm	Yes	No

Differences were calculated as the mean of the later morphology category minus the mean of the earlier category. Interobserver MDC95 values were 3.12° for SMA and 1.00 mm for SAS; intraobserver MDC95 values were 3.82° and 1.54 mm, respectively. SMA, superomedial angle; SAS, subacromial space; MDC95, minimum detectable change at the 95% confidence level.

**Table 5:** AUC-based discrimination of superomedial angle for pairwise acromial morphology comparison. Sample-derived Youden thresholds and the corresponding sensitivity, specificity, and positive and negative predictive values are exploratory and are provided in Supplementary Table S5.

Comparison	AUC (95% CI)
Type I vs. Type II	0.788 (0.724–0.848)
Type I vs. Type III	0.936 (0.891–0.972)
Type II vs. Type III	0.715 (0.647–0.779)

AUC, area under the curve; CI, confidence interval.

**Table 6:** Patient-level clustered GEE logistic regression models.

Outcome	Predictor	Adjusted OR (95% CI)	*p*-Value
Type III vs. Type I/II	SMA, per 1° increase	1.18 (1.11–1.25)	<0.001
Type III vs. Type I/II	SAS, per 1-mm increase	0.67 (0.52–0.86)	0.002
Type III vs. Type I/II	Age, per 1-year increase	1.02 (0.99–1.05)	0.137
Type III vs. Type I/II	Male sex	1.85 (0.98–3.47)	0.057
Type III vs. Type I/II	Right side	0.97 (0.56–1.69)	0.927
Type I vs. Type II/III	SMA, per 1° increase	0.77 (0.72–0.83)	<0.001
Type I vs. Type II/III	SAS, per 1-mm increase	1.75 (1.28–2.41)	<0.001
Type I vs. Type II/III	Age, per 1-year increase	1.02 (0.98–1.05)	0.347
Type I vs. Type II/III	Male sex	1.67 (0.79–3.52)	0.182
Type I vs. Type II/III	Right side	1.69 (0.83–3.43)	0.147

GEE, generalized estimating equation; OR, odds ratio; CI, confidence interval; SMA, superomedial angle; SAS, subacromial space.

**Table 7:** Ordinal logistic regression for rotator-cuff severity with patient-level cluster-robust standard errors. OR denotes odds ratio; CI, confidence interval; SMA, superomedial angle; SAS, subacromial space. Acromial type is modeled with Type I as the reference category.

Predictor	OR	95% CI	*p*
SMA (per 1°)	1.00	0.96–1.06	0.842
SAS (per 1 mm)	0.61	0.48–0.78	<0.001
Age (per 1 year)	1.07	1.05–1.10	<0.001
Male sex	1.11	0.69–1.80	0.664
Type II vs. Type I	1.74	0.75–4.04	0.196
Type III vs. Type I	1.22	0.46–3.28	0.690

## Data Availability

The data presented in this study are available on request from the corresponding author due to privacy and institutional restrictions on patient-level medical imaging data.

## Supplementary Materials

The following supporting information can be downloaded at: https://www.mdpi.com/article/10.3390/jcm15176612/s1, STROBE-Checklist; Table S1: interobserver agreement and weighted kappa for acromial classification between the two radiologists, including the full Radiologist 1 by Radiologist 2 cross-tabulation. Table S2: direct correlation between SMA and SAS overall and within acromial morphology categories. Table S3: SAS outlier sensitivity analysis after exclusion of shoulders with observer-mean SAS < 3 mm. Table S4: optimism-corrected (bootstrap) AUC estimates for pairwise SMA discrimination. Table S5: exploratory threshold-based diagnostic metrics (sensitivity, specificity, PPV, NPV) for SMA. Table S6: ordinal (proportional-odds) logistic regression of acromial morphology on SMA and SAS, with non-clustered and cluster-robust estimates. Table S7: sensitivity analysis of SMA and SAS across acromial morphology after exclusion of full-thickness rotator-cuff tears. Table S8: sensitivity analysis stratified by rotator-cuff status (normal versus any abnormality). Table S9: sensitivity analysis stratified by age (below versus at or above the median). Table S10: adult-only sensitivity analysis (patients aged 18 years or older). Table S11: superomedial angle in relation to rotator-cuff status, showing mean SMA by cuff severity within each acromion type (Panel A), within-type SMA versus cuff-severity correlations (Panel B), and Type II versus Type III discrimination in cuff-normal and cuff-abnormal shoulders (Panel C).
