# Supplementary material for: MRI-Derived Scapular Superomedial Angle and Acromial Morphology: A Retrospective Cross-Sectional Reliability and Morphometric Association Study
Source: J Clin Med. 2026 Aug 27;15(17):6612. doi: 10.3390/jcm15176612 (PMC13566210; doi:10.3390/jcm15176612)
Supplement: Supplementary file 1 [file jcm-15-06612-s001.zip › jcm-4444203-STROBE-Checklist.pdf]

STROBE Statement - Checklist of Items for Cross-Sectional Studies

MRI-Derived Scapular Superomedial Angle and Acromial Morphology: A Retrospective Cross-Sectional Reliability and Morphometric Association Study

The following checklist indicates where each STROBE item is addressed in the manuscript. Section numbers refer to the numbered headings of the submitted manuscript; table and figure numbers refer to the main text unless prefixed with S, which denotes the Supplementary Materials.

Section / Topic	Item	STROBE Recommendation	Location in Manuscript
Title and abstract	1	(a) Indicate the study's design with a commonly used term in the title. (b) Provide in the abstract an informative and balanced summary of what was done and what was found.	Title; Abstract ("retrospective cross-sectional MRI study"; structured Background/Methods/Results/Conclusions)
**Introduction**			
Background/rationale	2	Explain the scientific background and rationale for the investigation being reported.	Introduction, paragraphs 1-6
Objectives	3	State specific objectives, including any prespecified hypotheses.	Introduction, final two paragraphs (primary and secondary objectives)
**Methods**			
Study design	4	Present key elements of study design early in the paper.	Methods 2.1 (Study Design and Reporting); Abstract
Setting	5	Describe the setting, locations, and relevant dates, including periods of recruitment, exposure, follow-up, and data collection.	Methods 2.1-2.2 (single-center; Erzincan University, Mengücek Gazi TRH; MRI examinations May-June 2023)
Participants	6	(a) Give the eligibility criteria, and the sources and methods of selection of participants.	Methods 2.2 (Eligibility Criteria and MRI Protocol); Figure 1 (STROBE-style flow diagram)
Variables	7	Clearly define all outcomes, exposures, predictors, potential confounders, and effect modifiers.	Methods 2.3 (Image Assessment: SMA, SAS, acromial morphology); 2.5 (covariates: age, sex, side, rotator-cuff status)
Data sources/measurement	8	For each variable of interest, give sources of data and details of	Methods 2.2 (MRI protocol/scanner parameters); 2.3 (measurement algorithm, PACS tools, separate reader sets); 2.4

		methods of assessment (measurement). Describe comparability of assessment methods if there is more than one group.	(interobserver and intraobserver reliability methods)
Bias	9	Describe any efforts to address potential sources of bias.	Methods 2.3 (separate reader sets to minimize circularity; mutual blinding); 2.4 (blinded sessions); predefined retention of the senior radiologist's classification for discordant cases
Study size	10	Explain how the study size was arrived at.	Methods 2.1 (consecutive eligibility during predefined study period; precision interpreted via CI, SEM, MDC95 rather than a priori power)
Quantitative variables	11	Explain how quantitative variables were handled in the analyses. If applicable, describe which groupings were chosen and why.	Methods 2.5 (continuous SMA/SAS; acromial morphology as ordinal Type I–III; Type IV excluded with rationale in 2.2)
Statistical methods	12	(a) Describe all statistical methods, including those used to control for confounding. (b) Describe any methods to examine subgroups and interactions. (c) Explain how missing data were addressed. (d) If applicable, describe analytical methods taking account of sampling strategy. (e) Describe any sensitivity analyses.	Methods 2.4-2.5 (interobserver and intraobserver ICC, Bland–Altman, weighted kappa, SEM/MDC95; Kruskal-Wallis/post-hoc; pre-specified analysis hierarchy with clustered binary GEE as primary, proportional-odds ordinal model as supporting, and pairwise ROC with bootstrap as exploratory; rotator-cuff status modeled as ordinal (cluster-robust) and binary (GEE) outcomes adjusted for SMA, SAS, age, sex, and acromial type; sensitivity analyses incl. full-thickness-tear exclusion, RC-status and age strata, adult-only, and SAS <3 mm outlier exclusion) No missing values were present for any variable included in the primary or sensitivity analyses
**Results**			
Participants	13	(a) Report numbers of individuals at each stage of the study. (b) Give reasons for non-participation at each stage. (c) Consider use of a flow diagram.	Results 3.1; Methods 2.2 (screening 353 -> 306 with itemized exclusions); Figure 1 (flow diagram)
Descriptive data	14	(a) Give characteristics of study participants and information	Results 3.1; Table 1 (demographic and imaging characteristics)

		on exposures and potential confounders. (b) Indicate number of participants with missing data for each variable of interest.	
Outcome data	15	Report numbers of outcome events or summary measures.	Results 3.1-3.3; Table 1, Table 3 (acromial morphology distribution and SMA/SAS by type)
Main results	16	(a) Give unadjusted estimates and, if applicable, confounder-adjusted estimates and their precision. Make clear which confounders were adjusted for and why. (b) Report category boundaries when continuous variables were categorized. (c) If relevant, consider translating estimates of relative risk into absolute risk.	Results 3.2-3.6; Tables 2-7; interobserver and intraobserver reliability with 95% CI/LoA (Table 2); between-category differences vs MDC95 (Table 4); AUCs (Table 5); clustered GEE adjusted for age, sex, side (Table 6); rotator-cuff severity model (Table 7)
Other analyses	17	Report other analyses done - e.g., analyses of subgroups and interactions, and sensitivity analyses.	Results 3.3-3.6; Table 7 (rotator-cuff severity vs SMA/SAS/age/sex/acromial type); Supplementary Tables S2-S11 (full-thickness-tear exclusion, RC-status and age strata, optimism-corrected AUC, ordinal model, adult-only, correlation, SAS <3 mm outlier, and SMA by rotator-cuff status within acromion type)
**Discussion**			
Key results	18	Summarise key results with reference to study objectives.	Discussion, opening paragraph (three main findings)
Limitations	19	Discuss limitations of the study, taking into account sources of potential bias or imprecision. Discuss both direction and magnitude of any potential bias.	Discussion (slice-selection reproducibility not separable from landmark placement; MDC95 vs adjacent-category difference; kappa interpretation/external validity; SAS confounders; selection bias from clinically indicated MRI over-representing cuff-affected shoulders; degenerative variables not separately graded; Type IV exclusion)
Interpretation	20	Give a cautious overall interpretation of results considering objectives, limitations, multiplicity of	Discussion (SMA as complementary continuous descriptor, not a Bigliani surrogate or individual diagnostic threshold)

		analyses, results from similar studies, and other relevant evidence.	
Generalisability	21	Discuss the generalisability (external validity) of the study results.	Discussion (single-center, fixed-protocol context; need for multicenter, multireader, and prospective validation)
**Other information**			
Funding	22	Give the source of funding and the role of the funders for the present study and, if applicable, for the original study on which the present article is based.	Funding statement (no external funding)

Note. An Explanation and Elaboration article discusses each checklist item and gives methodological background and published examples of transparent reporting. The STROBE checklist is best used in conjunction with that article (freely available on the STROBE website: https://www.strobe-statement.org).
