# Supplementary material for: MRI-Derived Scapular Superomedial Angle and Acromial Morphology: A Retrospective Cross-Sectional Reliability and Morphometric Association Study
Source: J Clin Med. 2026 Aug 27;15(17):6612. doi: 10.3390/jcm15176612 (PMC13566210; doi:10.3390/jcm15176612)
Supplement: Supplementary file 1 [file jcm-15-06612-s001.zip › jcm-4444203-Supplementary Tables.pdf]

Supplementary Materials

MRI-Derived Scapular Superomedial Angle and Acromial Morphology: A Retrospective Cross-Sectional Reliability and Morphometric Association Study

Acromial morphology was classified independently by two radiologists. Table S1 presents the two independent initial readings used to quantify interobserver reliability. The two readings were concordant in 302 of 306 shoulders. For the four discordant shoulders, the classification of Radiologist 2, who was the senior reader, was retained as the final category. Consequently, the column totals in Table S1 correspond to the final classification used in all subsequent analyses: Type I, 51; Type II, 178; and Type III, 77. The row totals represent the independent classifications of Radiologist 1 before the final classification was assigned. All subsequent descriptive, receiver-operating-characteristic, generalized-estimating-equation, and sensitivity analyses in the main text and Supplementary Materials were based on the final classification.

Table S1. Interobserver agreement for acromial morphology classification between the two independent radiologist readings, with the full cross-tabulation.

Radiologist 1 (rows) \ Radiologist 2 (columns)	Type I	Type II	Type III	Row total
Type I	50	0	0	50
Type II	1	176	1	178
Type III	0	2	76	78
Column total	51	178	77	306

Observed agreement: 302/306 (98.7%). **Cohen kappa:** 0.977 (95% CI, 0.952-0.995). **Linear-weighted kappa:** 0.980 (95% CI, 0.960-0.995). **Quadratic-weighted kappa:** 0.984.

Note. Rows correspond to Radiologist 1 and columns to Radiologist 2. Confidence intervals for kappa were obtained by bias-corrected bootstrap resampling with 2000 replications. All four discordant classifications involved adjacent categories: one Type I–II disagreement and three Type II–III disagreements; no Type I–III disagreement occurred. Radiologist 2 was the senior reader, and their classification was retained as the final category for the discordant cases. Therefore, the column totals correspond to the final classification of Type I, 51; Type II, 178; and Type III, 77 used in all subsequent analyses, whereas the row totals represent the independent classifications of Radiologist 1 before final classification was assigned.

Table S2. Direct correlation between superomedial angle and subacromial space, overall and within acromial morphology categories.

Group	n	Pearson r	Spearman rho
Overall cohort	306	-0.03 (p = 0.607)	-0.10 (p = 0.095)
Type I	51	0.12 (p = 0.396)	0.15 (p = 0.302)
Type II	178	0.14 (p = 0.066)	0.11 (p = 0.162)
Type III	77	0.07 (p = 0.526)	0.03 (p = 0.801)

Note. Pearson and Spearman correlation coefficients between superomedial angle and subacromial space. The overall correlation was negligible, and within-type correlations were weak and non-significant, with point estimates slightly positive rather than negative. Simple linear regression of subacromial space on superomedial angle confirmed the absence of a meaningful linear relationship (beta = -0.007 mm per degree; p = 0.607; R-squared < 0.001). These results indicate that superomedial angle and subacromial space vary in opposite directions across acromial morphology categories but are not directly correlated with each other at the individual level.

Table S3. SAS outlier sensitivity analysis after exclusion of shoulders with observer-mean SAS <3 mm (n = 303).

Variable	Type I (n = 51)	Type II (n = 175)	Type III (n = 77)	p value
SMA, °	128.7 ± 4.2	134.0 ± 5.3	137.2 ± 4.4	<0.001
SAS, mm	7.5 ± 1.2	6.9 ± 1.1	6.3 ± 1.2	<0.001

Clustered multivariable models after exclusion of SAS <3 mm outliers.

Outcome	Predictor	Adjusted OR (95% CI)	p value
Type III vs Type I/II	SMA, per 1° increase	1.18 (1.11-1.25)	<0.001
Type III vs Type I/II	SAS, per 1-mm increase	0.60 (0.46-0.78)	<0.001
Type I vs Type II/III	SMA, per 1° increase	0.77 (0.72-0.83)	<0.001
Type I vs Type II/III	SAS, per 1-mm increase	1.64 (1.15-2.33)	0.006

Note. Values are mean ± standard deviation; p values are from Kruskal-Wallis tests. Three shoulders had observer-mean SAS <3 mm; all were Type II acromia with full-thickness rotator-cuff tears. Clustered GEE models were adjusted for age, sex, and side. Findings were unchanged in direction and significance, indicating that extremely low SAS values did not drive the main SAS-related findings.

Table S4. Apparent and bootstrap optimism-corrected discrimination (AUC) of superomedial angle for pairwise acromial morphology comparison.

Comparison	Apparent AUC	Optimism	Optimism-corrected AUC
Type I vs Type II	0.788	<0.001	0.788
Type I vs Type III	0.936	<0.001	0.936
Type II vs Type III	0.715	0.001	0.714

Note. Discrimination was based on the observer-mean superomedial angle and the retained final acromial classification, identical to the main-text receiver-operating-characteristic analysis; the apparent AUC values therefore match Table 5 of the main text. Optimism was estimated by bootstrap resampling with 2000 replications. Because discrimination relied on a single continuous predictor without fitted model parameters, optimism was negligible and the corrected estimates were essentially identical to the apparent values.

Table S5. Exploratory threshold-based diagnostic metrics of superomedial angle for pairwise acromial morphology discrimination.

Comparison	Youden cutoff	Sensitivity	Specificity	PPV	NPV
Type I vs Type II	≥134.0°	47.8%	96.1%	97.7%	34.5%
Type I vs Type III	≥134.5°	75.3%	100.0%	100.0%	72.9%
Type II vs Type III	≥135.5°	72.7%	67.4%	49.1%	85.1%

Note. Thresholds were derived within the same sample using the Youden index and are exploratory only. PPV and NPV are positive and negative predictive values, which depend on the sample prevalence of each morphology and are not externally validated diagnostic values. These metrics are provided for completeness and must not be used as clinical decision cutoffs. The corresponding area-under-the-curve discrimination is reported in Table 5 of the main text.

Table S6. Ordinal (proportional-odds) logistic regression of acromial morphology (Type I < Type II < Type III) on superomedial angle and subacromial space, with non-clustered and cluster-robust estimates.

Predictor	Odds ratio (95% CI)	Cluster-robust OR (95% CI)	p value
SMA, per 1° increase	1.22 (1.17-1.28)	1.22 (1.16-1.29)	<0.001
SAS, per 1-mm increase	0.61 (0.51-0.74)	0.61 (0.48-0.78)	<0.001

Note. Proportional-odds model fitted by maximum likelihood on the retained final classification. The "Odds ratio (95% CI)" column reports the conventional non-clustered model, and the "Cluster-robust OR (95% CI)" column reports the same model with sandwich standard errors accounting for patient-level clustering of the 27 bilateral cases. The proportional-odds (parallel-lines) assumption was examined by comparing category-specific coefficients; the direction of association was consistent across cut-points, although some variation in coefficient magnitude was observed. Higher superomedial angle and lower subacromial space were associated with more hooked acromial morphology under both specifications, and the close agreement between the non-clustered and cluster-robust estimates indicates that the bilateral cases did not materially affect the inference. These results reproduce the direction and significance of the binary models in the main text. Superomedial angle and subacromial space were not collinear (Pearson r = -0.03), arguing against over-adjustment when both were included in the same model. The cluster-robust estimates were computed in R using sandwich-type cluster-robust covariance accounting for patient-level clustering; the non-clustered model is reported as the primary supporting ordinal analysis alongside the main-text cluster-robust generalized-estimating-equation models.

Table S7. Sensitivity analysis of superomedial angle and subacromial space across acromial morphology after exclusion of full-thickness rotator-cuff tears (n = 288).

Variable	Type I (n = 49)	Type II (n = 167)	Type III (n = 72)	p value
SMA, °	128.7 ± 4.2	134.1 ± 5.4	137.2 ± 4.4	<0.001
SAS, mm	7.6 ± 1.0	6.9 ± 1.1	6.4 ± 1.0	<0.001

Note. Values are mean ± standard deviation. Group comparisons by Kruskal-Wallis test. Eighteen shoulders with full-thickness (complete) rotator-cuff tears were excluded. The stepwise SMA increase and SAS decrease across acromial morphology persisted.

Table S8. Sensitivity analysis stratified by rotator-cuff status.

Rotator-cuff-normal subgroup (n = 76)

Variable	Type I (n = 20)	Type II (n = 42)	Type III (n = 14)	p value
SMA, °	129.4 ± 4.2	133.6 ± 5.8	135.8 ± 4.6	<0.001
SAS, mm	7.7 ± 1.3	7.1 ± 1.0	6.3 ± 0.9	0.002

Any rotator-cuff abnormality subgroup (n = 230)

Variable	Type I (n = 31)	Type II (n = 136)	Type III (n = 63)	p value
SMA, °	128.1 ± 4.1	134.0 ± 5.3	137.6 ± 4.3	<0.001
SAS, mm	7.3 ± 1.2	6.7 ± 1.4	6.3 ± 1.3	<0.001

Note. Values are mean ± standard deviation. Group comparisons by Kruskal-Wallis test. Any rotator-cuff abnormality was defined as tendinosis, partial-thickness tear, or full-thickness tear. The subgroup totals sum to the full analytic cohort (51/178/77), and the SMA and SAS gradients across acromial morphology were preserved within both subgroups.

Table S9. Sensitivity analysis stratified by age (median split at 50 years).

Age below 50 years (n = 152)

Variable	Type I (n = 29)	Type II (n = 91)	Type III (n = 32)	p value
SMA, °	128.6 ± 4.0	132.9 ± 5.5	136.7 ± 4.1	<0.001
SAS, mm	7.7 ± 0.9	6.8 ± 1.1	6.4 ± 1.0	<0.001

Age 50 years or older (n = 154)

Variable	Type I (n = 22)	Type II (n = 87)	Type III (n = 45)	p value
SMA, °	128.8 ± 4.5	134.9 ± 5.1	137.6 ± 4.6	<0.001
SAS, mm	7.2 ± 1.5	6.8 ± 1.6	6.2 ± 1.4	0.002

Note. Values are mean ± standard deviation. Group comparisons by Kruskal-Wallis test. The strata sum to the full analytic cohort (51/178/77). The SMA and SAS gradients were preserved within both age strata, indicating the associations are not explained by age alone.

Table S10. Adult-only sensitivity analysis of superomedial angle and subacromial space across acromial morphology, restricted to patients aged 18 years or older (n = 305).

Variable	Type I (n = 51)	Type II (n = 177)	Type III (n = 77)	p value
SMA, °	128.7 ± 4.2	133.8 ± 5.3	137.2 ± 4.4	<0.001
SAS, mm	7.5 ± 1.2	6.8 ± 1.3	6.3 ± 1.2	<0.001

Note. Values are mean ± standard deviation. Group comparisons by Kruskal-Wallis test. One skeletally immature patient (aged 15 years) was excluded. The stepwise superomedial-angle increase and subacromial-space decrease across acromial morphology were essentially unchanged compared with the full cohort, indicating that inclusion of patients younger than 18 years did not drive the observed associations.

Table S11. Superomedial angle in relation to rotator-cuff status, and stability of Type II versus Type III discrimination by cuff status.

**Panel A. Mean superomedial angle (°) by rotator-cuff severity within acromion type**					
**Acromion type**	**Normal**	**Tendinosis**	**Partial-thickness**	**Full-thickness**	**p***
Type I	129.4 ± 4.2 (20)	128.0 ± 4.4 (17)	128.2 ± 4.2 (12)	128.5 ± 4.2 (2)	0.691
Type II	133.6 ± 5.8 (42)	133.2 ± 4.3 (55)	135.0 ± 5.8 (70)	131.4 ± 4.6 (11)	0.101
Type III	135.8 ± 4.6 (14)	137.1 ± 4.2 (25)	137.8 ± 4.6 (33)	137.8 ± 4.2 (5)	0.506
**Panel B. Within-type correlation between superomedial angle and cuff severity**					
**Acromion type**	**Spearman ρ**	**p**			
Type I	−0.152	0.287			
Type II	+0.101	0.182			
Type III	+0.168	0.143			
**Panel C. Type II versus Type III discrimination by cuff status**					
**Subgroup**	**AUC**	**Youden threshold (°)**	**n**		
Cuff-normal	0.691	134.5	56		
Cuff-abnormal	0.721	136.0	199		

*Values are mean ± SD (n) unless otherwise stated. *Kruskal-Wallis test across cuff-severity levels. No statistically significant within-type association between SMA and recorded rotator-cuff severity was detected. The exploratory Type II versus Type III thresholds were broadly similar between cuff-normal and cuff-abnormal subgroups; however, no formal interaction analysis was performed, and residual degenerative, positional, and imaging-related confounding cannot be excluded.*
